# “Molecular Docking and Dynamic Studies of Amide Derivatives from Cinnamic Acid with Potential Anti‐Dengue Virus Activity”

**DOI:** 10.1002/open.202500107

**Published:** 2025-07-16

**Authors:** Luis Alfonso Cárdenas‐Granados, Manuel Alejandro Hernández‐Serda, Omar Joel Villegas‐Solís, Víctor Hugo Vázquez‐Valadez, Aldo Yoshio Alarcón‐López, Pablo A. Martínez‐Soriano, Jaqueline Ramos‐Sánchez, Tannya Karen Castro‐Jiménez, José Bustos‐Arriaga, Enrique Angeles

**Affiliations:** ^1^ Laboratorio de Química Medicinal y Teórica FES Cuautitlán Universidad Nacional Autónoma de México (UNAM) Campo 1 Av. 1 de Mayo SN Cuautitlán Izcalli Mexico City 54740 Mexico; ^2^ Departamento de Ciencias Químicas FES Cuautitlán Universidad Nacional Autónoma de México (UNAM) Av. 1 de Mayo SN Cuautitlán Izcalli Mexico City 54740 Mexico; ^3^ Departamento de Ciencias Biológicas FES Cuautitlán Universidad Nacional Autónoma de México (UNAM) Av. 1 de Mayo SN Cuautitlán Izcalli Mexico City 54740 Mexico; ^4^ QSAR Analytics S.A. de C.V. Témpano 10 Colonia Atlanta Cuautitlán Izcalli Estado de México CP 54740 Mexico; ^5^ Laboratorio de Biología Molecular e Inmunología de arbovirus Unidad de Biomedicina Facultad de Estudios Superiores Iztacala Universidad Nacional Autónoma de México Tlalnepantla 54090 México

**Keywords:** dengue, molecular dynamics, virtual screening, viruses

## Abstract

Dengue, classified as a neglected tropical disease and transmitted by *Aedes* mosquitoes, remains a significant global health challenge, often evolving into severe clinical manifestations such as hemorrhagic fever. Despite its widespread impact, no antiviral therapy has been approved to date, highlighting the urgent need for effective and accessible treatment options. In the present work, computational analysis is performed on an in‐house library of easily synthesized caffeic acid phenethyl ester analogs, which exhibit potential activity against the viral envelope (E) protein, a critical mediator of dengue virus entry and membrane fusion. Among them, LQM778 demonstrated consistent stability within the protein–ligand complex during molecular dynamics simulations. This finding provides a foundation for in vitro studies and future structural optimizations that could transform the landscape of antiviral development against dengue.

## Graphical abstract

A library of easily synthesizable CAPE analogs was screened *in silico* against the dengue virus envelope protein. Compound LQM778 showed stable binding and promising inhibitory potential. This study highlights LQM778 as a candidate for future in vitro validation and structural optimization toward accessible antiviral agents against dengue.

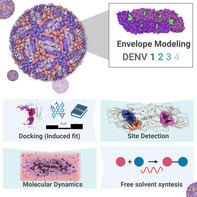

## 1 Introduction

Neglected tropical diseases (NTDs) encompass various infectious diseases that affect a billion people in 149 countries, particularly underdeveloped regions.^[^ ^1^ ^]^ These diseases predominantly affect low‐ and middle‐income populations with limited access to healthcare services.^[^ ^2^ ^]^ The WHO identifies 20 main NTDs. NTDs affect more than 10% of the global population, especially poor and indigenous people.^[^ ^3^ ^]^

**Figure 1:**
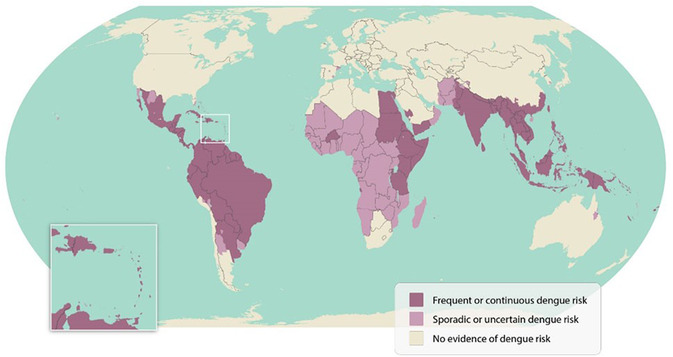
Country reporting cases (Figure obtained April 2025).^[^ ^48^ ^]^

**Figure 2:**
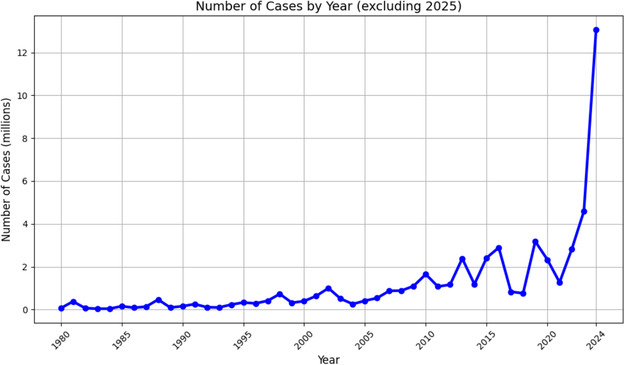
Dengue cases affecting countries throughout the Americas over time (data obtained April 2025).^[^ ^49^ ^]^

The transmission of arboviruses such as dengue and chikungunya remains a major public health problem in Africa, Latin America, and the Caribbean. Unfortunately, surveillance and control measures in these regions are not consistent and, in some cases, may not be sensitive enough to detect low levels of transmission. As shown in **Figure** **1** and **2**, the number of dengue cases progressively increased over time, affecting multiple countries.

The genus Flavivirus includes a group of arboviruses, which are viruses transmitted by vectors and belong to the family Flaviviridae. These viruses include single‐stranded RNA (ssRNA+) with positive polarity and a length of ≈11 kb. Its distinctive replication mechanism classifies it within group IV of the Baltimore classification.^[^ ^4^, ^5^, ^6^ ^]^

**Figure 3:**
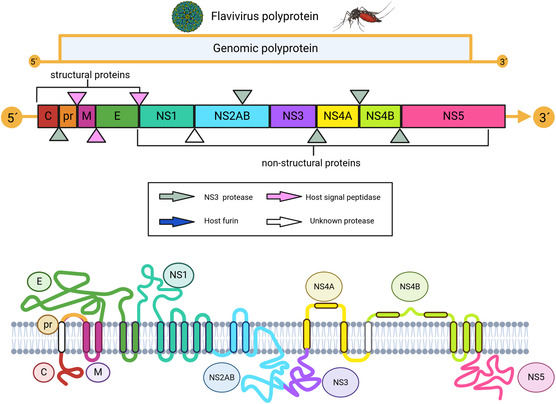
Flavivirus polyprotein. The flavivirus genome encodes a polyprotein that is cleaved into different functional proteins. Different proteases perform specific cleavages in polyproteins.

In this group of viruses, there are viruses wrapped with a capsid that is 37 to 50 nm wide, and surface proteins arranged in an icosahedral shape. The genetic material of the virion plays a role in both the genome and the messenger RNA (**Figure** **3**).^[^ ^7^, ^8^ ^]^

When these viruses genomes are translated, a polyprotein is generated. This protein is then processed by viral and host proteases both during and after translation. This process leads to the formation of 3 structural proteins (C, prM, and E) and 7 nonstructural proteins. (NS1, NS2A, NS2B, NS3, NS4A, NS4B, and NS5).^[^ ^5^, ^6^, ^9^, ^10^ ^]^

We focused our attention on the importance of protein E, as it is responsible for receptor binding and possesses fusogenic properties. Furthermore, this protein determines both tropism and binding specificity.^[^ ^11^ ^]^

The first domain (DI), comprising segments 1–52, 133–193, and 281–296, occupies a central position in the 3D structure between domains II (DII) and III (DIII), playing a critical role in protein stability and functioning as an essential hinge bridge.^[^ ^6^, ^12^ ^]^

**Figure 4:**
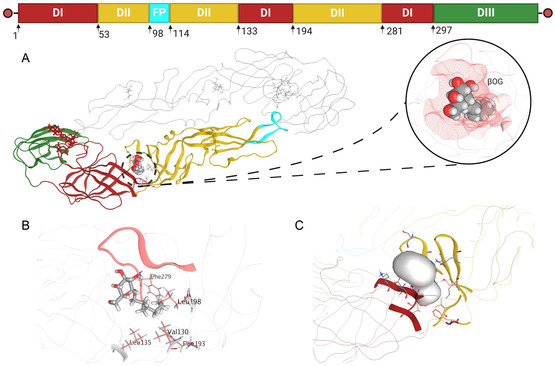
Dimer domains of pE. A) Folding of glycoprotein E (PDB:1OKE). B) Important amino acids at the β‐OG site. C) Site search by SiteFinder.

Amino acids from segments 53–132 and 194–280 make up the DII domain. Within this domain is the fusion peptide (FP), which is located between amino acids 98–113 (**Figure** **4**). Different flaviviruses typically conserve at least 75% of these amino acids. Researchers have reported that three hydrophobic amino acids (W101, L107, and F108) in FPs are essential for the integration of the E protein trimer into the endosomal membrane and the successful execution of fusion between the viral membrane and the endosomal membrane.^[^ ^9^, ^13^ ^]^

A hydrophobic spot called the “β‐OG pocket” was found in studies of the 3D structure. This is where N‐octyl‐D‐glucoside (N‐β‐OG or β‐OG) is stored. This area, which is very similar to that of other flaviviruses, is thought to be a key spot for E protein entry inhibitors. It also changes shape and functions as a hinge through a glycine zone. (residues 268–280).^[^ ^14^ ^]^

This area may be very important in the infection cycle because it affects shape and allows pH to affect the fusion peptide.

Domain III (DIII) has a structure similar to an immunoglobulin and is composed of ≈100 amino acids (residues 297–394). It is an important epitope for the immune system and a place where receptors can bind. In the interaction with the receptor, the important amino acids are K305, K307, K310, V382, and G385.^[^ ^5^, ^13^, ^15^, ^16^, ^17^, ^18^ ^]^

**Figure 5:**
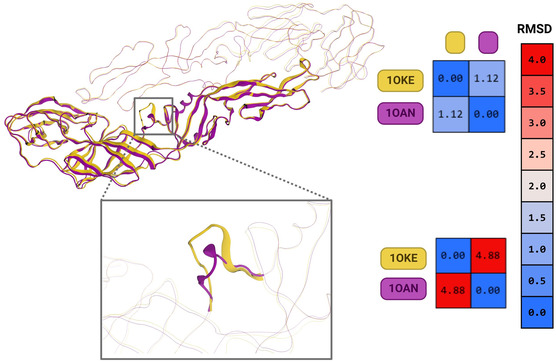
Structural overlay of protein E (1OKE yellow) (1OAN purple). RMSD global top. The RMSD of the lower part of the β‐OG site.

The part of the protein where the N‐β‐OG ligand binds has a fork‐shaped shape that can open when a ligand is present but usually stays closed when it is not present. The root‐mean‐square deviation (RMSD) values were found to be low when all the alpha carbons of the E protein were compared. This was seen by superimposing the structures of the protein data bank (PDB) entries 1OKE and 1OAN, which are both from DENV2. However, comparing only residues 268–280 revealed a significantly greater RMSD in this region (**Figure** **5**).

Recently, significant advances have been made in the development of antiviral drugs, with a particular focus on specific proteins from various viruses. Researchers worldwide have employed advanced molecular modeling techniques to discover compounds with potential antiviral effects on flaviviruses. These studies provide important structural information about how viral proteins interact with organic molecules that can prevent them from functioning.^[^ ^16^, ^17^ ^]^

Researchers have shown that flaviviruses can enter host cells by fusing viral and cellular membranes together. This is accomplished by the viral envelope protein E, which changes shape when the pH level in the endosomal compartment of host cells decreases. Scientists have investigated how small molecules can target certain parts of viral proteins. For example, researchers have investigated the hydrophobic pocket of the chikungunya virus capsid protein and the dimer interface of the Japanese encephalitis virus E protein.^[^ ^18^ ^]^

These advances offer hope in the fight against viral infections, especially with the recent dengue and zika epidemics and the ongoing COVID‐19 pandemic. Instead of targeting general antiviral agents, which can harm healthy cells, antiviral agents can target specific viral proteins.

The discovery of organic molecules that can combat flaviviruses has led to significant advances. Several notable studies have been conducted in this field, such as research on compound P02 as an antiviral agent, (S)‐(+)‐mandelic acid (MDA), and ethyl 3‐aminobenzoate in the context of the chikungunya virus, which is also transmitted by *Aedes aegypti*.^[^ ^19^, ^20^ ^]^ Additionally, four FDA‐approved drugs, tretinoin, mefenamic acid, ondansetron, and artemether, have been identified as potential inhibitors of ion channels formed by the SARS‐CoV‐2 E protein.^[^ ^21^ ^]^ Hexamethylene amiloride has also shown promise in blocking E‐channel activity.^[^ ^22^ ^]^ Furthermore, researchers have developed peptides that target the West Nile virus envelope protein and show promise for use in antiviral strategies.^[^ ^10^ ^]^ In addition, there are reports of the use of natural products, such as Amustaline, for the treatment of these viral diseases, as well as other studies.^[^ ^23^, ^24^, ^25^, ^26^, ^27^ ^]^

Similarly, amides are among the chemical groups that have shown intriguing biological properties. Numerous studies have reported their antiviral activity, highlighting their potential to inhibit different viruses. These investigations suggest that amides can interfere with the viral replication cycle and the interaction between the virus and the host cell. Authors from different works have documented these effects, emphasizing the importance of amides in the development of new antiviral treatments.^[^ ^28^, ^29^, ^30^, ^31^, ^32^, ^33^, ^34^ ^]^

**Figure 6:**
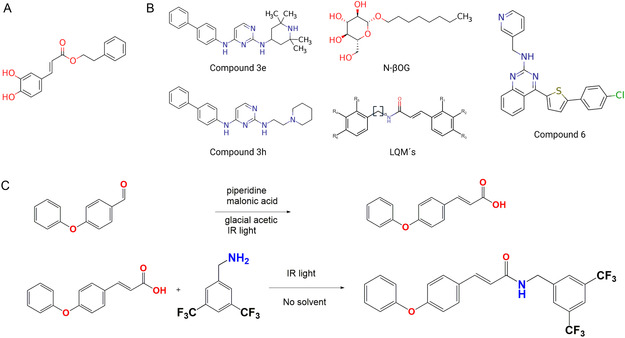
A) Structure of CAPE. B) Chemical structures of the compounds used in molecular docking, as well as the base scaffold of the LQM 700 series. C) Synthesis outline for LQM778.

Studies were conducted to propose small molecules as potential specific antivirals against dengue. Based on this premise, our research group investigated the LQM 700 series, a group of cinnamic acid derivatives. These chemicals are made from the structure of the phenethyl ester of caffeic acid (CAPE), which is found in propolis (**Figure** **6**A). Propolis is a natural substance that has many biological effects, such as fighting viruses, bacteria, and fungi and improving the immune system.^[^ ^30^, ^31^, ^32^, ^33^, ^34^ ^]^

As part of these studies, the compound with the best antiviral activity is being synthesized and tested. The goal is to make dengue treatments more specific and effective.

The LQM700 series is composed of cinnamic acid analogs and derivatives. The amide derivatives of cinnamic acids are very chemically stable because they do not break down easily with enzymes and do not have any esterase substrates. This feature makes amide derivatives particularly promising for the development of antiviral agents.

## 2 Experimental Section

### 2.1 *In Silico* Studies

Representative amino acid sequences of pE proteins from DENV serotypes 1–4 were identified using the Basic Local Alignment Search Tool protein (BLASTp) from the National Center for Biotechnology Information (NCBI), allowing the retrieval of protein sequences corresponding to different dengue virus serotypes.^[^ ^35^ ^]^ Shared binding sites among DENV serotypes were identified, and sequence similarity percentages were calculated to guide the selection of modeling templates (DENV1–4) from the PDB.^[^ ^36^ ^]^

The GenBank genetic sequences were translated into amino acid sequences via the ExPASy Translate Tool from the Swiss Institute of Bioinformatics; all the data are shown in **Table** **1**.^[^ ^37^, ^38^, ^39^ ^]^

**Table 1:** Access codes to PDB structures or sequences used.

VIRUS	PDB ID	UNIPROT CODE
DENV1	N/A	P17763
DENV2	1OAN/1OKE	GENBANK:FJ390389.1 P12823
DENV3	N/A	P27915
DENV4	N/A	P09866

### 2.2 Protein Modeling

The crystallographic structure corresponding to PDB ID: 1OKE was selected as the template for modeling the envelope (E) protein across different DENV serotypes. This structure was specifically chosen to promote the opening of the binding pocket, a feature particularly relevant to the insertion of a ligand of the E protein. Homology modeling was conducted using Swiss‐Modeler, incorporating sequences from the DENV1, DENV3, and DENV4 serotypes prior to pocket opening (Figure S1.1, Supporting Information)^[^ ^40^ ^]^

Model validation was rigorously conducted via the Swiss‐Modeler assessment server, with particular scrutiny on stereochemical integrity, the minimization of atomic clashes, and the reduction of outliers within the Ramachandran distribution.

Subsequent structure preparation was performed in MOE 2022, with a concerted emphasis on the accurate modeling of glycosylation motifs, highly conserved and functionally indispensable in DENV pathogenesis, as well as the manual incorporation of disulfide bridges absent from initial predictions. Protonation states were assigned using the “Protonate 3D” utility within MOE, calibrated to physiological conditions (pH 7.0, 300 K), to ensure biologically relevant electrostatic profiles.^[^ ^41^ ^]^

Thereafter, the modeled structures underwent sequential alignment and pairwise structural superposition.

All the images were generated via molecular software, such as MOE, Chimera X, VMD, and Biorender.^[^ ^41^, ^42^, ^43^, ^44^ ^]^

### 2.3 Ligand Modeling

An in‐house library from the LQM700 series, consisting of cinnamic acid derivatives, was employed for the analysis. This library had four experimental compounds that worked against the dengue virus in a reported pocket: N‐βOG, compound 3e, compound 3 h, and compound 6, in addition to these ligands (Figure 6B).^[^ ^5^ ^]^

Ligands were prepared by first importing their SMILES codes into a database. They were then processed via a washing procedure to ensure the correct protonation states and geometries. A conformational search was subsequently performed via the LowModeMD method included in the MOE toolset. The search was limited to 10 000 conformations, with an RMS gradient of 0.005.

### 2.4 Molecular Docking

On the other hand, the pockets were analyzed by SiteFinder from MOE tools and the FPocketWeb server, and the β‐OG pocket was selected as a promising site (Figure S2.1, Supporting Information).^[^ ^45^ ^]^

The docking protocol was conducted in two steps. First, a placement step was performed via the triangle matcher algorithm, generating a total of 1000 poses, which were scored via the London dG scoring function. In the second step, refinement was carried out via the induced fit method, with the binding modes evaluated via the GBVI/WSA dG scoring function. Validation of the protocol was conducted using the N‐ β‐OG in the original model for DENV2 (PDB:1OKE) (Figure 2.3, Supporting Information). For each ligand, the top binding modes were retained for further analysis.

To analyze the residues affected by the presence of a ligand bound to the protein, we used the ProDy ESSA tool, employing a total of 10 normal modes with Gaussian Normal Modes (GNMs).^[^ ^46^ ^]^

### 2.5 Molecular Dynamics Simulations

Molecular dynamics simulations were performed for both APO and HOLO forms of the DENV1 E protein under periodic boundary conditions, using explicit water for solvation and charge neutralization with Na^+^ and Cl^−^ ions. A particle mesh Ewald correction of 1 Å was applied, with an equilibration phase comprising a 100 ps NVT ensemble followed by a 100 ps NPT ensemble, and a production run of 500 ns. Simulations were carried out at 300 K and 100 kPa using the AMBER12:EHT force field implemented in NAMD, with the APO state and the complexes formed by the top‐ranked ligand from virtual screening as well as the reference ligand N‐βOG.^[^ ^47^ ^]^

Protein–ligand interactions were evaluated at 200 ns of the simulation to determine the most common interactions between the complexes and hydrogen bonds between the ligands and the E protein over 500 ns.

### 2.6 Synthesis of LQM778

Through computer simulations, we synthesized LQM778 (Figure 6C), which was the best scoring compound. The analogs exhibit a wide range of success rates, which helps us to learn more about the structural features that make them effective against viruses. This knowledge can then be used to guide future improvements and the creation of better treatments.

#### 2.6.1 Synthesis of 3‐(4‐Phenoxyphenyl)‐2‐Propenoic Acid

We weighed 1 g of 4‐phenoxybenzaldehyde and 1 g of malonic acid in a round‐bottom flask. We then added piperidine (1 mL, 11.76 mmol) and glacial acetic acid (2.5 mL, 41.66 mmol). We heated the mixture under IR light at 130–140 °C for 1 h. After this period, we cooled the reaction mixture and added ice‐cold water to induce precipitation. We allowed the flask to return to room temperature and filtered and washed the precipitate with room‐temperature water until no traces of aldehyde remained. We added an adequate amount of ethyl acetate to dissolve the crystals and used anhydrous sodium sulfate to remove any water that had been absorbed. Recrystallization yielded white needle‐shaped crystals.

#### 2.6.2 Synthesis of (E)‐N‐(3,5‐bis(trifluoromethyl)benzyl)‐3‐(4‐Phenoxyphenyl)Acrylamide (LQM778)

We placed 3‐(4‐phenoxyphenyl)‐2‐propenoic acid and 3,5‐bis(trifluoromethyl)benzylamine in a flask and connected it to a reflux apparatus at a 1:1 molar ratio. We heated the mixture at 140 to 160 °C using a 300 W IR light bulb. We performed thin‐layer chromatography using hexane/acetate (50:50) to monitor the progress of the reaction. Upon completion, we added sufficient ethyl acetate to dissolve the reaction product. The reaction mixture was mixed with a small amount of activated charcoal. The charcoal was then removed by filtering the mixture through diatomaceous earth (Hyflo Super Cel from Sigma–Aldrich). The corresponding amide was formed by recrystallization from ethyl acetate.

## 3 Results and Discussion

### 3.1 Protein Modeling

Multiple sequence alignment of the envelope (E) proteins from the four dengue virus serotypes revealed a relatively high degree of sequence conservation, with overall identity exceeding 65%. Although crystallographic structures are available in public repositories for various DENV serotypes, the majority depict the E protein in a more conformationally restricted, closed state. This structural limitation impedes the exploration of the binding site of interest, particularly in serotypes for which no experimentally resolved structures with an open pocket are available.

To overcome this limitation, homology models were generated for DENV1, DENV3, and DENV4 using a structure favoring pocket accessibility (PDB ID: 1OKE) as a template. These models were thoroughly evaluated and subsequently refined for downstream molecular docking studies. The resulting ensemble exhibited a remarkable degree of conformational conservation, as reflected by a mean RMSD of merely 0.154 Å. (Figure S1.2, Supporting Information)

### 3.2 Molecular Docking

Based on the results obtained from site identification tools, and taking into account ligand‐binding information from the crystallographic structure PDB:1OKE, site 3 consistently highlighted across both predictive approaches was selected as the target region for molecular docking (Section 2, Supporting Information).

Docking simulations were carried out to assess binding affinities and explore optimal binding poses, with evaluation guided by the scoring function implemented in MOE. As reported in **Table** **2**, flexible docking analysis revealed that compound LQM778 exhibits a favorable binding profile, suggesting a strong potential for interaction with the selected pocket.

**Table 2:** Scoring in DENV 1.

Compound	Score [kcal mol^−1^]	LE
3 h	−7.95	0.27
3e	−7.77	0.28
778	−7.74	0.23
Compound 6	−7.54	0.25
735	−7.52	0.27
768	−7.52	0.28
769	−7.35	0.25
755	−7.35	0.27
764	−7.2	0.27
753	−7.16	0.28

To contextualize these findings, we benchmarked the binding affinities of known β‐OG site ligands, including N‐β‐octylglucoside (N‐βOG) and compounds 3e, 3h, and 6, alongside a series of in‐house derivatives from the LQM700 compound library. Ligand efficiency (LE) metrics were also computed, providing a normalized measure of binding performance that accounts for structural variation among the ligands (Figure S2.2, Supporting Information).

**Figure 7:**
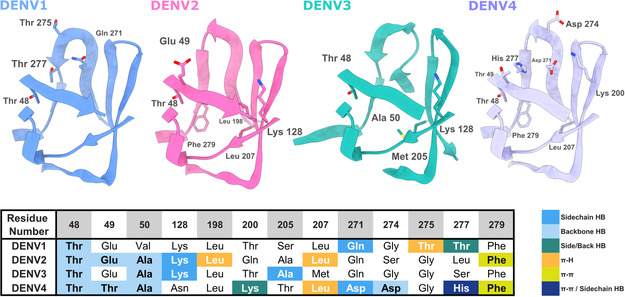
Main Interactions of LQM778 with the 4 serotypes of DENV.

Analyzing the interactions observed in the binding poses of the LQM778 ligand with the target site in the four dengue virus serotypes, we found that hydrogen bonds are commonly formed in the upper region of the pocket in most serotypes (**Figure** **7**). These interactions primarily involve the backbone of residues 48–50. Another notable interaction, although not present in all four serotypes, is with the conserved residue Phe279. In addition, a hydrogen bond is formed with Gln271. These findings are consistent with the characteristics of the pocket, where the deepest region tends to favor more hydrophobic interactions.

To gain insights into the dynamic behavior of the E protein upon ligand binding, we performed a Gaussian Normal Modes (GNM) analysis using both the reference ligand N‐β‐octylglucoside (N‐βOG) and the top‐scoring compound identified during virtual screening, LQM778. This approach enabled us to evaluate residue‐level fluctuations and assess the allosteric impact of ligand occupation within the binding pocket.

**Figure 8:**
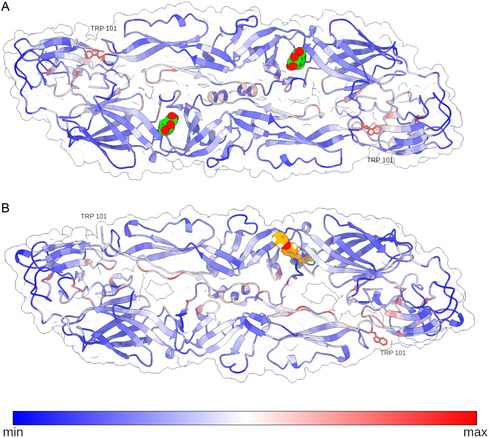
A) GNM of pE with the presence of a N‐BOG ligand bound in the βOG pocket. B) GNM of pE with the presence of LQM778 bound in the βOG pocket.

As depicted in **Figure** **8**, the GNM results revealed distinct alterations in residue flexibility upon binding of LQM778, particularly in regions proximal to the binding site. Notably, one of the most perturbed residues was TRP101, a conserved residue located within the fusion peptide, an element essential for membrane fusion and viral entry in flaviviruses. The observed modulation of TRP101 dynamics underscores the potential of LQM778 to interfere with key steps in the DENV replication cycle through stabilization or perturbation of this functionally critical region.

Compound LQM778 was found to adopt two distinct binding orientations within the identified pocket. In the first binding mode, both trifluoromethyl (CF_3_) groups are directed toward the interior of the protein, engaging residues at the base of the pocket. In contrast, the second binding mode features the CF_3_ groups oriented outward, facing the exterior surface of the virion. Notably, the “CF_3_‐up” orientation, where the trifluoromethyl groups are exposed toward the virion surface, emerged as the predominant pose across most dengue virus serotypes, as illustrated in Figure S2.4, Supporting Information.

### 3.3 Molecular Dynamics Simulations

**Figure 9:**
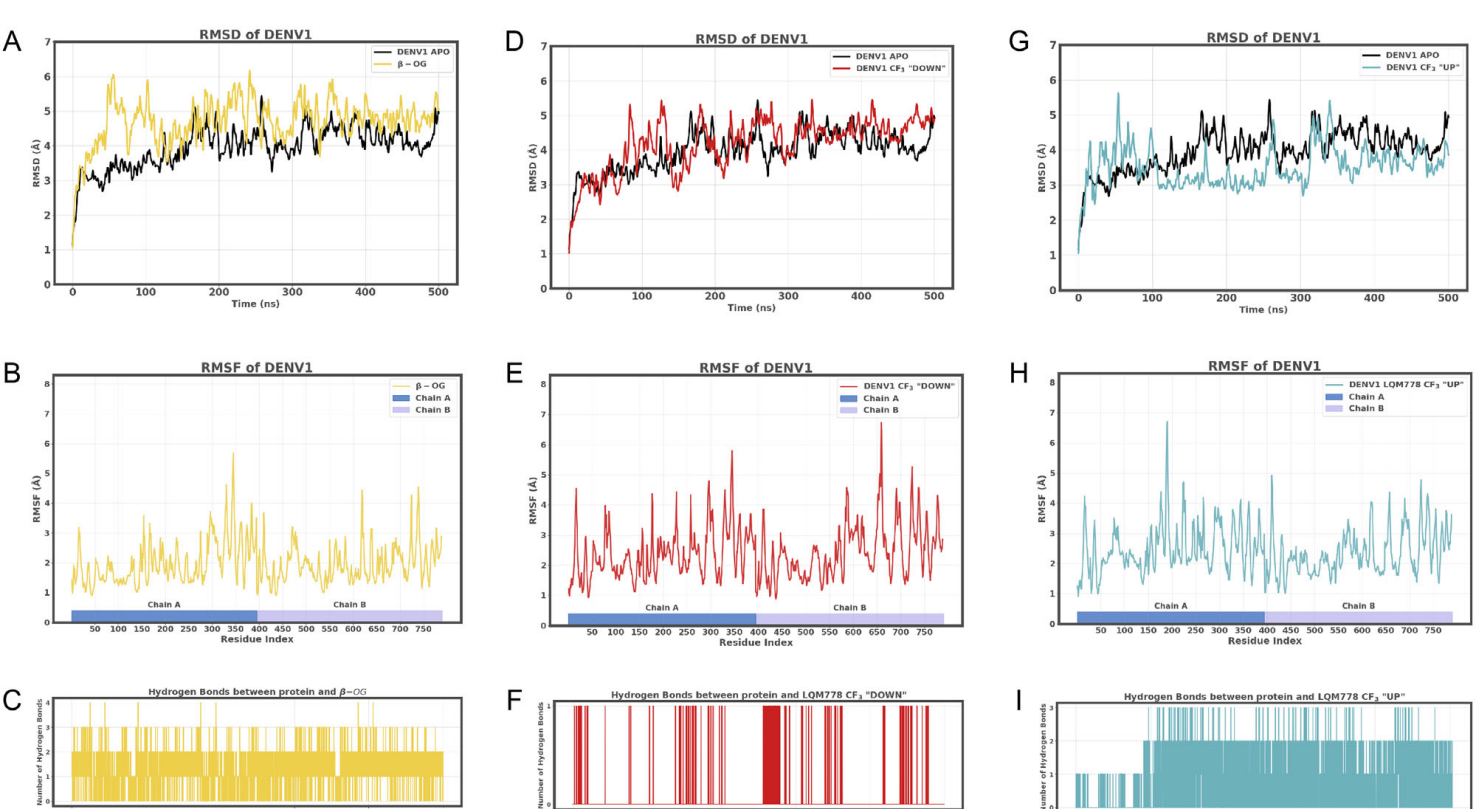
A,D,G) RMSD of the complex simulation of DENV1 pE and the N‐βOG ligand (yellow), LQM778 with CF_3_ groups oriented “down” (red), LQM778 with CF_3_ groups oriented “up” (blue), and the DENV1 pE APO state (black). B,E,H) RMSF of the complexes pE and ligands N‐βOG (yellow), LQM778 CF_3_ “down” (red), and LQM778 CF_3_ “up” (blue). C,F,I) Number of hydrogen bonds between pE and the ligands N‐βOG (yellow), LQM778 CF_3_ “down” (red), and LQM778 CF_3_ “up” (blue).

The β‐OG pocket of the E protein was selected as the target site due to its hydrophobic nature and its potential role in modulating the viral entry process; the molecular dynamics of the DENV1 APO form were analyzed to identify highly flexible regions and to explore the conformational behavior of the E protein in search of cryptic pockets, such as the β‐OG site evaluated (**Figure** **9**).

The molecular dynamics simulations presented in Figure 8A, D,G demonstrate that the pE–ligand complexes remain structurally stable over the course of 500 ns, with the ligands consistently retained within the β‐OG binding site. While the reference ligand N‐β‐octylglucoside (N‐β OG) is capable of forming up to six hydrogen bonds, the LQM778 ligand, particularly in its “CF_3_‐up” orientation, maintains its stability through the formation of up to three persistent hydrogen bonds.

RMSD values for all ligand‐bound systems closely mirror those of the ligand‐free (APO) simulation, indicating that ligand binding does not compromise the overall structural integrity of the E protein. Interestingly, the BOG ligand exhibits multiple binding modes during the simulation, occasionally forming up to four hydrogen bonds. Figure 9F illustrates that when LQM778 adopts the “CF_3_‐down” orientation i.e., with the trifluoromethyl groups directed toward the pocket interior, only a single hydrogen bond is formed throughout the simulation. Conversely, Figure 9I depicts the alternative “CF_3_‐up” pose, wherein the CF_3_ groups are oriented toward the exterior surface of the envelope protein.

Further analysis of root‐mean‐square fluctuations (RMSF) revealed an increase in local flexibility within residues 190–197 at the base of the β‐OG pocket upon LQM778 binding, relative to the baseline fluctuations observed in the APO chains. This suggests localized conformational perturbations induced by ligand interaction.

**Figure 10:**
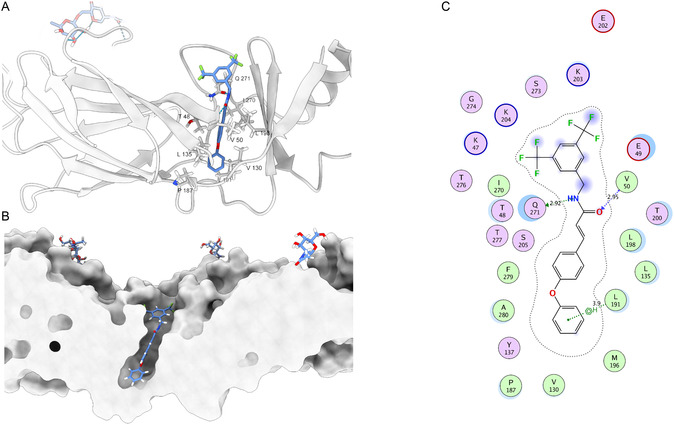
A) Important interactions of 3D LQM778 in the E protein–ligand complex at 200 ns of simulation. B) Occupancy of the pocket in the protein by the ligand. C) 2D interactions between the E protein and the ligand LQM778.

As shown in **Figure** **10**, the lower region of the binding pocket is predominantly stabilized through hydrophobic interactions. In contrast, the upper region accommodates key polar interactions formed by LQM778, including hydrogen bonds with V50 and Q271, as well as a π–hydrogen interaction with L191, collectively contributing to the ligand's retention and orientation within the site.

**Figure 11:**
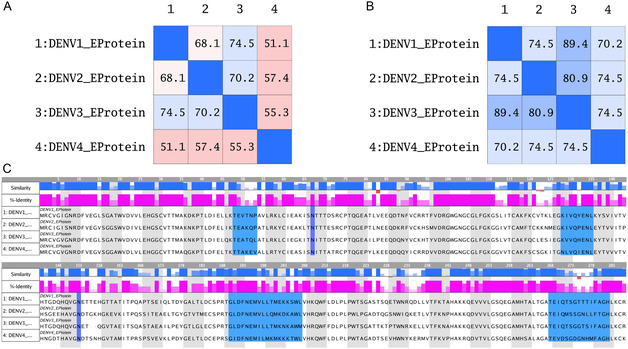
A) Identity matrix of 4 Dengue envelope protein serotypes in the β‐OG pocket. B) Similarity matrix of 4 Dengue envelope protein serotypes in the β‐OG pocket. C) Alignment of 4 serotypes of the dengue E protein; the β‐OG pocket is shown in blue, and the glycosylation sites are shown in purple, N67, and N153.

The region of the β‐OG pocket shows a degree of identity between the 4 serotypes of at least 51% identity (**Figure** **11**A) and high similarity between serotypes dengue 1 and dengue 3 of 89% identity (Figure 11B). L191 and Q271 are conserved in DENV1‐3 and interact in complex with LQM778.

**Figure 12:**
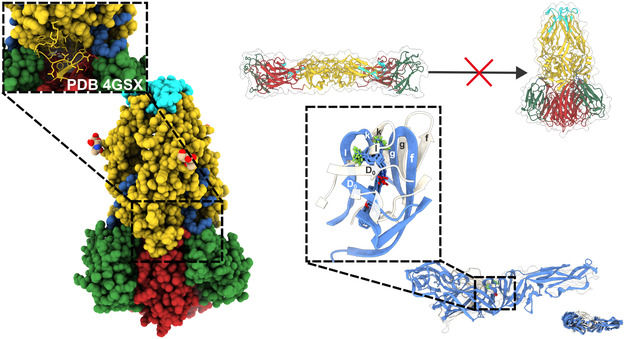
Superposition of the trimeric and dimeric forms of the DENV1 envelope protein obtained from the last 100 ns of the molecular dynamics simulation.

The crystallographic structure PDB ID: 4GSX, which corresponds to the trimeric form of the DENV1 envelope (E) protein, exhibits the β‐OG binding pocket in a closed conformation. In contrast, the structural superposition of this trimer with representative frames extracted from the final 100 ns of the molecular dynamics simulations reveals pronounced conformational deviations, particularly within the *k* and *l* regions of the pocket and the adjacent D_0_ domain. Notably, the presence of the LQM778 ligand appears to play a key role in maintaining the pocket in an open conformation, with the most significant structural displacement observed in the *k* and *l* regions (**Figure** **12**). These observations suggest a potential ligand‐induced stabilization of an open‐state architecture, which may have implications for accessibility and binding dynamics within this allosteric site. (Figure 12).

### 3.4 Spectroscopy of LQM778

Spectroscopy of LQM 778. Rend. 68%, mp 140–142 °C ; ^1^H NMR (400 MHz, CDCl_3_, *δ*): 4.68 (2H, d, NH), 6.35 (1H, d, *J* = 15.6), 6.96 (2H, d), 7.03 (2H, d), 7.15 (1 H, t), 7.25 (1H, s), 7.36 (2H, t), 7.46 (2H, d), 7.66 (1H, d, *J* = 15.5), 7.77 (2H, d)^; 13^C NMR (100 MHz, CDCl_3_, *δ*): 42.83 (CH2‐Arom), 118.09, 118.41 (Arom), 119.61 (–CH=), 124.06 (CF3 JC‐F = 274.6 Hz), 121.46, 127.77, 129.16, 129.56, 129.53, 129.90, 131.78, 132.12 (Arom), 141.08 (–CH=), 141.74, 156.11, 159.26 (Arom), 166.16 (–CO–); IR (diamond, νmax, cm−1): 1509 (C=O), 3050 (CH Csp2), 3272 (NH), 1124 (C‐F); EIMS (*m*/*z* (%)): 108 (20) [*M* ^+^], 107 (60) [*M* ^+^ − H], 91 (100) [C H ^+^]; HRMS (ESI, *m/z*): [*M* + H]^+^ calcd for C_21_H_38_N_4_O_6_S, 475.2591; found, 475.2593. Anal. calcd. for C_24_H_17_F_6_NO_2_: C, 61.9; H, 3.6; N, 3.0 Found C, 62.12; H, 2.64; N, 3.43.

## 4 Conclusion

The envelope (E) protein of all four dengue virus serotypes was modeled using the crystallographic structure PDB:1OKE as a template. Site analysis revealed a highly druggable pocket, corresponding to the one identified in the 1OKE structure. Flexible docking simulations demonstrated that the ligand LQM778 exhibited binding affinities on par with previously reported active compounds, and notably surpassed the binding affinity of the crystallized reference ligand, N‐β‐octylglucoside (BOG).

Analysis of the binding pocket revealed that the upper region favors the formation of hydrogen bonds, while the middle and lower regions are stabilized through π–π and π–H interactions. To assess the effects of ligand binding on protein dynamics, Gaussian Normal Modes (GNM) analysis was conducted. While some perturbations were noted in residues associated with the fusion peptide, no significant conformational shifts were detected over the course of the 500 ns simulation when compared to the ligand‐free (APO) form.

These results suggest that ligand binding in the β‐OG pocket could disrupt the dimer–to–trimer transition, potentially inhibiting the closure of the *k–l* and *g–f* regions critical for viral fusion. Additionally, the orientation of the CF_3_ groups in the “up” position (toward the pocket exterior) appears to be the most favorable binding mode, likely due to the bulky nature of these groups, which helps stabilize the pocket in its open conformation. A notable interaction observed during the simulation was the hydrogen bond between Gln271 and the amide hydrogen of LQM778.

Further in vitro studies are required to confirm the antiviral efficacy of LQM778. However, the compound's straightforward two‐step synthesis makes it an attractive candidate for drug development, especially for regions severely affected by the dengue virus. Future studies should also focus on optimizing the pharmacokinetic (ADME) absortion, distribution, metabolism and excretion properties of LQM778 and similar ligands to enhance their drug‐like characteristics.

## Conflict of Interest

The authors declare no conflict of interest.

## Supporting information

Supplementary Material

## Data Availability

The data that support the findings of this study are available from the corresponding author upon reasonable request.
