# Supplementary material for: “Molecular Docking and Dynamic Studies of Amide Derivatives from Cinnamic Acid with Potential Anti‐Dengue Virus Activity”
Source: ChemistryOpen. 2025 Jul 16;14(11):e202500107. doi: 10.1002/open.202500107 (PMC12598814; doi:10.1002/open.202500107)
Supplement: Supplementary file 1 — Supplementary Material [file OPEN-14-e202500107-s001.pdf]

Supporting Information

1.Protein Modeling

To compare, we obtained models of the same Dengue virus serotype 2 envelope protein using AlphaFold as shown in Figure 1. Although the models show a relatively low RMSD when superimposing all residues of the E protein (below 3 Å), in the region corresponding to the "BOG" pocket (residues 48–53, 128–135, 190–207, and 268–282), they exhibit a higher RMSD compared to the PDB structure 1OAN, which corresponds to a closed conformation of the BOG site.

Moreover, AlphaFold predicts this region with low confidence, as indicated by the pLDDT scores.

When specifically analyzing the fg and kl regions (residues 196–213 and 269–281, respectively), we observed that AlphaFold models fail to fully open the pocket of interest.

Finally, the SWISS-MODEL server was selected to perform the Homology modeling.

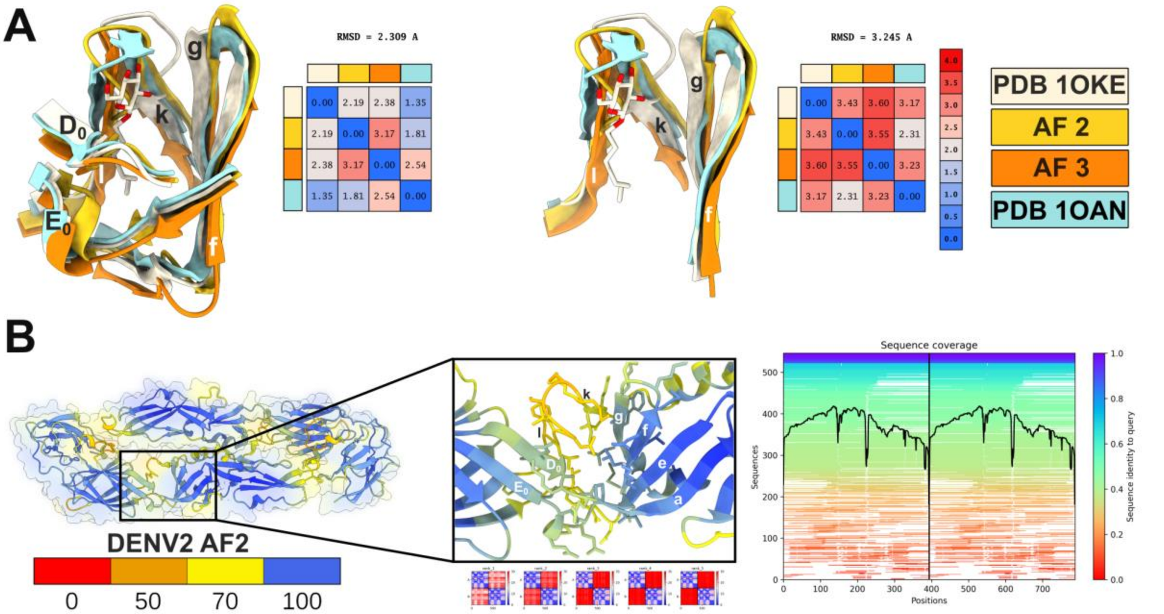

Figure 1.1 Superposistion of DENV2 E proteins model comparing with the closed state (PDB:1OAN) and the open state (PDB:1OKE)(A). Protein modeling for Envelope Protein of DENV serotype 2 using Alphafold (B),

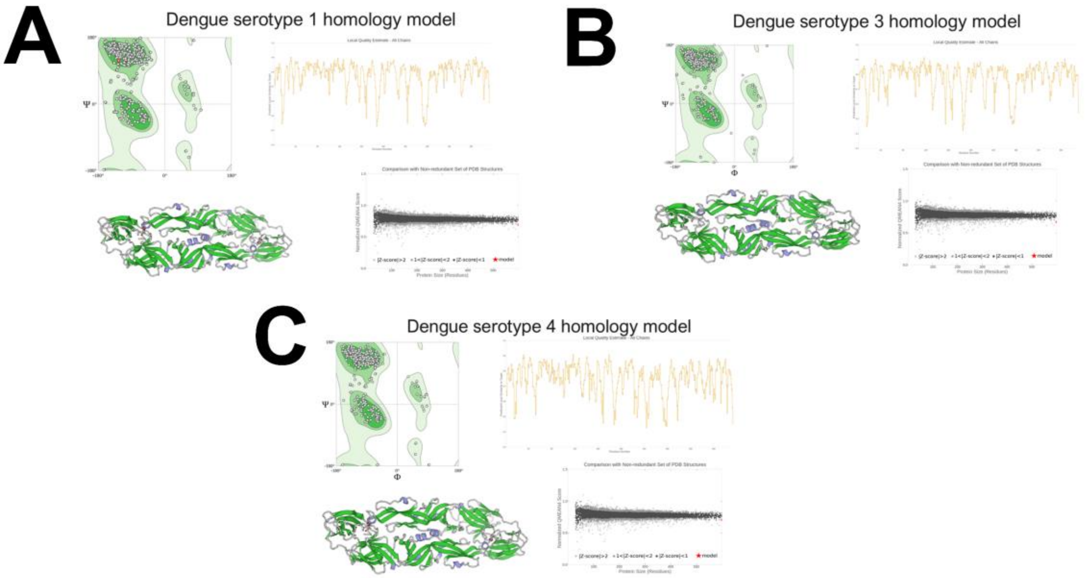

Figure 1.2 A) Results of homology modeling of DENV1, DENV3 and DENV4 (A-C) with Ramachandran plot, local quality estimate, QMEAN4 score and final model.

The final models of the Envelope dengue protein using the PDB:1OKE model as template successfully open the cryptic site essential for the virtual screening process Figure 1.2.

In most cases the Ramachandran plots show a majority of favored amino acids with a few outliers and A decent local quality estimation.

2. Molecular Docking

Using the MOE tool SiteFinder and Fpocketweb we keep the top 3 sites according to binding scores or drugabbility scores as shown in Figure 2.1, since both site prediction methods ranked this pocket in top three places, and considering the previous data from the PDB structure 1OKE, this same pocket was selected for molecular docking. Tables 2.1-3 shows the information of the evaluated pockets and the residues involved in.

To perform the docking, a set was created within the MOE software the specific residues selected for molecular docking were: Glu26, Lys47–Pro53, Val130, Ans134–Tyr137, Met196–Gln200, Asp203–Val208, Gly190–Phe193, and Thr268–His282.

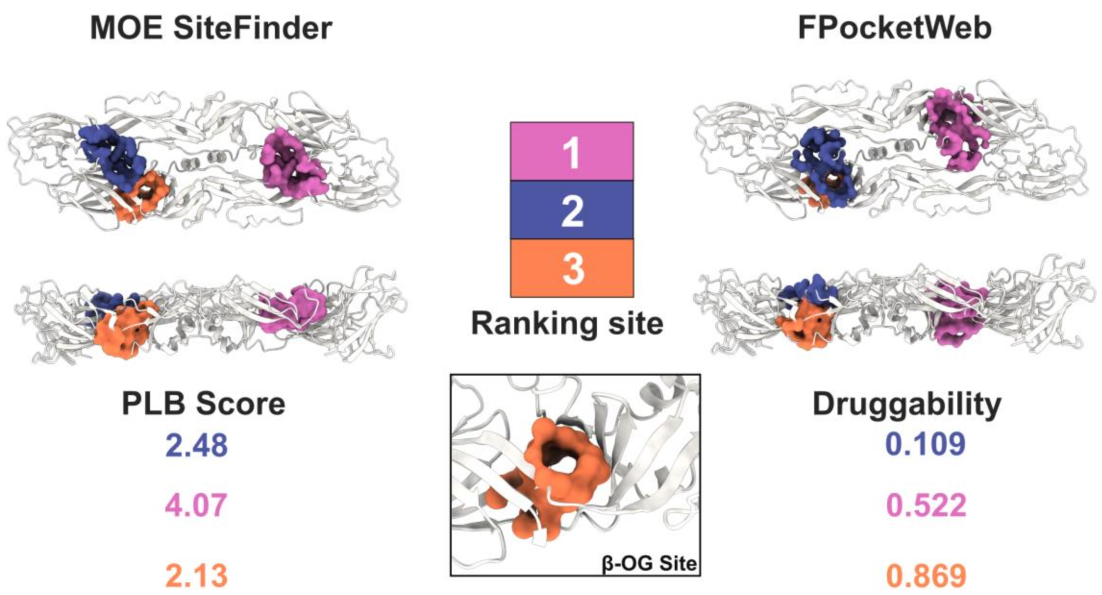

Figure 2.1 Top 3 SiteFinder and Fpocket results.

Table 2.1 Site Finder MOE Results

MOE Site Finder (Molecular probe: 1.4 and 1.8)

Site	Number of Alpha Spheres	PLB	Hydrophobic contacts	Sidechain Contacts
1	150	4.07	34	65
2	120	2.48	27	52
3	105	2.13	43	52

Table 2.2 Residues for top 3 Sites Site Finder MOE

MOE Site Finder Residues

Site	**Residues**
1	1:(ARG2 CYS3 ILE4 GLY5 ILE6 SER7 HIS27 GLY28 SER29 GLU44 LEU45 ILE46 LYS47 VAL151 GLY152 ASP154 GLY275 ASN276 LEU277 LEU278)2:(VAL97 ASP98 ARG99 GLY100 TRP101 GLY102 ASN103 PHE108 ASN242 PRO243 HIS244 ALA245 LYS246 LYS247)
2	1:(ASP98 ARG99 GLY100 TRP101 GLY102 ASN103 PHE108 HIS244 ALA245 LYS246 LYS247)2:(ARG2 CYS3 ILE4 GLY5 ILE6 SER7 HIS27 GLY28 SER29 GLU44 LEU45 ILE46 LYS47 VAL151 GLY152 ASP154 GLY275 ASN276 LEU277)
3	2:(THR48 ALA50 VAL130 PRO132 LEU135 TYR137 PRO166 SER186 PRO187 ARG188 THR189 GLY190 LEU191 PHE193 LEU198 GLN200 ALA205 LEU207 ILE270 GLN271 LEU277 PHE279 THR280 GLY281 HIS282 LEU283 LYS284)

Table 2.3 Fpocketweb Results

Fpocketweb Results

Site	Number of Alpha Spheres	Druggability	Hydrophobicity score	Volume Score
1	200	0.522	23	3.957
2	116	0.109	13	3.933
3	88	0.869	49	4

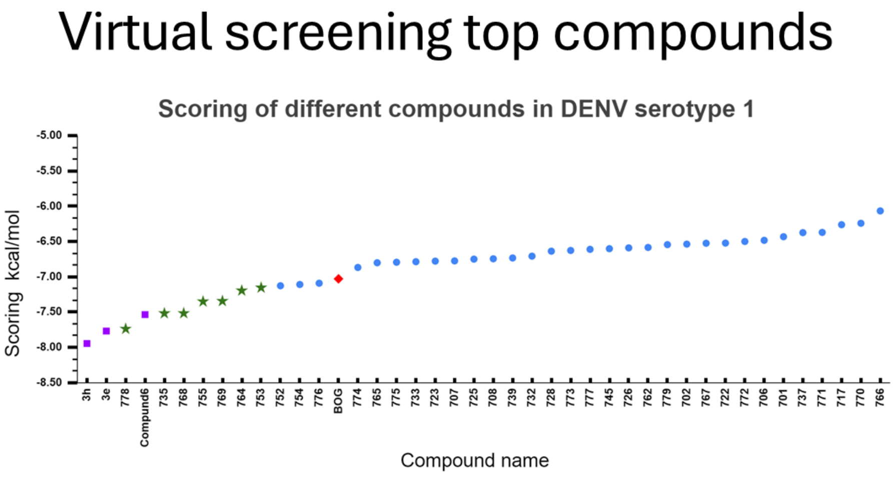

Figure 2.2 Top scoring compounds of virtual screening process.

The top scored compounds in the virtual screening process of the LQM700s library and compounds with antiviral activity against Dengue tested *in vitro.*

The top 7 *in house* compounds LQM778, LQM735, LQM768, LQM755, LQM769, LQM764, LQM763 shows best affinity energy in the scoring function used than the co-crystallized ligand in PDB:1OKE (N-β-OG), additionally LQM778 presents similar values with de Compound 3h and Compound 3e, overcoming compound 6.

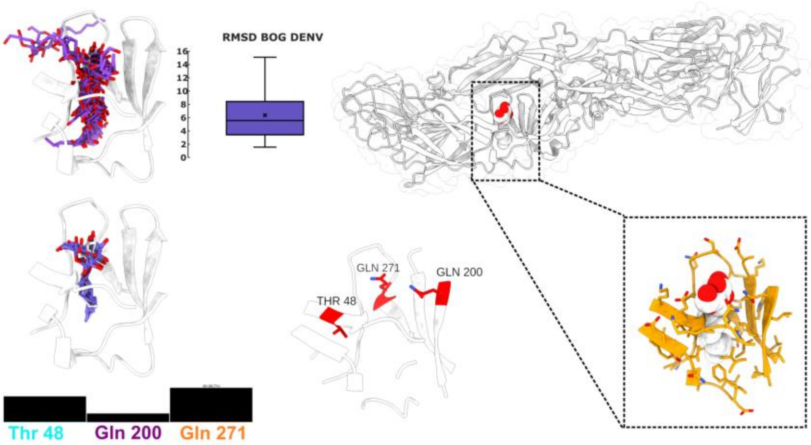

Figure 2.3. Redocking of PDB 1OKE showing the full cluster of binding modes and the binding modes with RMSD less than 3.0 A comparing with crystallized pose.

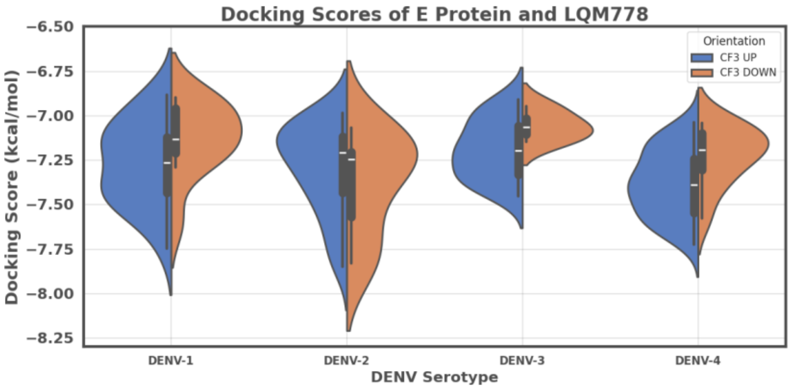

Figure 2.4. Distribution LQM docking scores in different Dengue Serotypes.

Best ligand 778 shows two well defined binding modes in the BOG pocket, with the principal difference in the orientation of CF3 groups, while in most serotypes DENV1, DENV3 and DENV4 the distribution aim to present a higher number of well scored binding modes with CF3 “up” in the case of DENV2 surprisingly the distribution of both binding poses are nearly symmetric distributions.

3. Molecular Dynamics

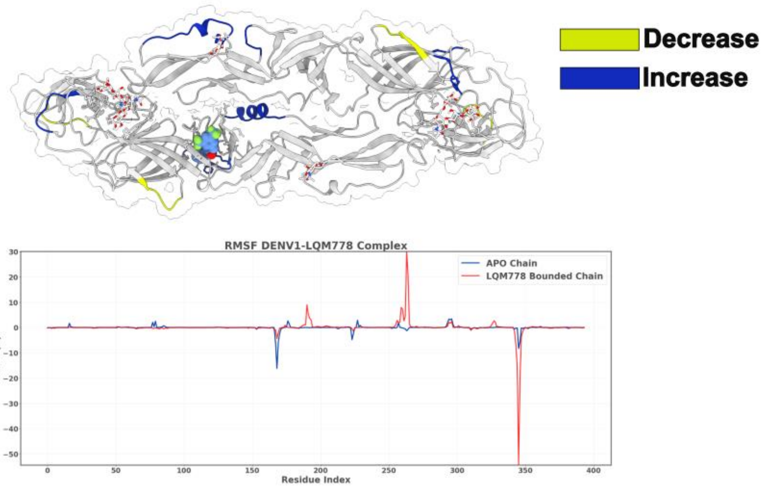

Figure 3. Most affected residues in HOLO simulation with LQM778 CF_3 “UP”

To show the most affected residues in their fluctuation along the simulation the two chains of the homodimer DENV1 APO simulation was averaged to act as baseline, then the difference between the APO baseline and the chains in the DENV1 complex (ligand bound and ligand unbound) were obtained.
